# Cancer-Associated Thrombosis in Breast Cancer: Risk Factors and Personalized Management

**DOI:** 10.3390/jcm15031161

**Published:** 2026-02-02

**Authors:** Sergey Kozhukhov, Nataliia Dovganych, Olha Lygyrda, Ivan Smolanka, Anton Loboda, Sergii Lyalkin

**Affiliations:** 1 State Institution “National Scientific Center” The M.D. Strazhesko Institute of Cardiology, Clinical and Regenerative Medicine of the National Academy of Medical Sciences of Ukraine, Kyiv 03151, Ukraine; dovganychnat@gmail.com; 2 Cardio-Oncology Center, Kyiv 03151, Ukraine; 3 National Cancer Institute, Kyiv 03022, Ukraine; olgalygyrda@gmail.com (O.L.); cardio.oncology.ukraine@gmail.com (I.S.); natadovhanych22@gmail.com (A.L.); slyalkin@yahoo.com (S.L.)

**Keywords:** breast cancer, prevention, risk factors, treatment, venous thromboembolism

## Abstract

**Background/Objectives:** Venous thromboembolism (VTE) is a major cardiovascular complication in cancer patients and the leading cause of morbidity and mortality. The aim of the study was to evaluate the incidence, timing, clinical predictors, and management of VTE in patients with breast cancer (BC), undergoing oncological therapy, and to propose a risk-adapted strategy for thrombosis monitoring and prevention. **Methods:** In this retrospective single-center study, 116 women with histologically confirmed BC (stages I–IV) treated between 2021 and 2024 were included. Patients were divided according to the occurrence of objectively confirmed VTE. Clinical characteristics, comorbidities, laboratory parameters, cancer-related factors, and treatment modalities were analyzed. Univariate and multivariate logistic regression analyses were performed to identify independent predictors of VTE. **Results:** VTE occurred in 25 patients (21.6%), predominantly within the first 12 months after cancer diagnosis. Patients who developed VTE were significantly older and more frequently had hypertension, dyslipidemia, hyperglycemia, anemia, and leukocytosis. Multivariate analysis identified age ≥ 55 years, poor performance status (ECOG ≥ 3), and elevated glucose level as independent predictors of VTE. Deep vein thrombosis of the lower and upper extremities was the most common manifestation (52%), while pulmonary embolism was present in 24% of cases, either alone or in combination (20%). Direct oral anticoagulants were the most frequently used long-term anticoagulant therapy. **Conclusions:** VTE is a clinically relevant and relatively frequent complication in patients with BC, particularly during the early period of anticancer treatment. Patient-related and metabolic factors play a key role in thrombosis risk, underscoring the need for individualized, risk-adapted approaches to VTE prevention and monitoring in these populations.

## 1. Introduction

Venous thromboembolism (VTE), encompassing deep vein thrombosis (DVT) and pulmonary embolism (PE), represents one of the most common and potentially life-threatening complications in patients with cancer. Cancer-associated thrombosis (CAT) is responsible for significant morbidity, increased healthcare utilization, and interruption of oncological therapy, and remains a leading cause of non-cancer-related mortality in cancer patients. The prothrombotic state in cancer is multifactorial and results from complex interactions between tumor biology, host-related factors, and anticancer treatments [1].

Breast cancer (BC) is the most frequently diagnosed malignancy among women worldwide and in Ukraine as well, and is associated with continuously improving survival due to advances in early detection and systemic therapy [2]. Historically, breast cancer has been categorized as a malignancy with a relatively low risk of VTE, particularly when compared with gastrointestinal, pancreatic, or lung cancers. Consequently, routine pharmacological thromboprophylaxis is not generally recommended for most patients with BC in current clinical guidelines. However, emerging evidence from population-based registries and real-world cohorts suggests that the risk of VTE in BC patients may be higher than previously assumed, especially during periods of active treatment [3,4].

Several factors may contribute to the development of VTE in BC patients, including advanced age, comorbid cardiovascular and metabolic conditions, reduced functional status, and cancer stage progression. In addition, modern treatment strategies, such as chemotherapy (CT), endocrine therapy, radiation therapy (RT), and surgical intervention, may further increase thrombotic risk through endothelial injury, systemic inflammation, and alterations in coagulation pathways [5]. Importantly, conventional risk stratification tools, such as the Khorana Risk Score, have demonstrated limited predictive value in BC population, highlighting the need for alternative or refined risk assessment approaches [6,7,8,9].

The early identification of patients at risk of VTE is essential to guide individualized prevention strategies and to balance the benefits of thromboprophylaxis against the risk of bleeding. From a cardio-oncology perspective, comprehensive cardiovascular assessment and close monitoring are particularly relevant, as thrombotic complications often coexist with other cardiovascular comorbidities and may adversely affect overall prognosis [10].

Therefore, the present study aimed to assess the incidence, timing, clinical characteristics, and predictors of VTE in patients with BC undergoing cancer treatment in a real-world setting. Additionally, based on the identified risk factors, we proposed a risk-adapted approach for thrombosis monitoring and prevention to support personalized management in this patient population.

## 2. Materials and Methods

### 2.1. Study Design and Participants

This retrospective, single-center observation study included 116 consecutive patients with BC (stages I–IV) who underwent cancer treatment between 2021 and 2024. All patients received therapy in accordance with current guidelines and protocols at the National Cancer Institute of the Ministry of Health of Ukraine. Patients were referred for cardiovascular evaluation and monitoring to the Cardio-Oncology Center at the State Institution National Scientific Center “The M.D. Strazhesko Institute of Cardiology, Clinical and Regenerative Medicine of the National Academy of Medical Sciences of Ukraine”, Kyiv, Ukraine.

Inclusion criteria: Adult patients (≥16 years) with histologically confirmed BC; availability of complete medical records; at least 12 months of follow-up. Exclusion criteria: History of VTE before cancer diagnosis; concurrent malignancies; chronic anticoagulation therapy at baseline; incomplete or missing key clinical data.

The median follow-up duration was 24 months (IQR: 21–28 months).

This study was conducted in compliance with the Declaration of Helsinki, and the study protocol was approved by the local institutional ethics committee, and all data were analyzed anonymously.

### 2.2. Study Endpoint and Group Stratification

The primary endpoint of the study was the occurrence of objectively confirmed VTE during the follow-up period. Based on the presence or absence of VTE, patients were divided into two groups: Group 1: patients who developed VTE during follow-up (*n* = 25), Group 2: patients without VTE events (*n* = 91).

### 2.3. Definition and Diagnosis of VTE

VTE included DVT of the upper or lower extremities, PE, including asymptomatic PE, and combined PE and DVT. Upper extremity DVT included thrombosis of the axillary, subclavian, and internal jugular veins. Lower extremity DVT involved thrombosis of the iliac, femoral, popliteal, fibular, and tibial veins. Peripheral DVT was confirmed using compression ultrasonography of the affected limb veins. PE was diagnosed by contrast-enhanced computed tomography.

### 2.4. Clinical and Cardiovascular Assessment

Patients’ comprehensive clinical and cardiovascular evaluation, along with detailed medical history, cardiovascular risk factors, and comorbidities, cancer-related characteristics (stage, histology, treatment modalities, CT regimens, and cumulative doses, RT exposure, surgical interventions) were performed.

Assessment of functional status using Eastern Cooperative Oncology Group performance status (ECOG PS), physical examination, 12-lead electrocardiography, and transthoracic echocardiography. Left ventricular ejection fraction (LV EF) was measured using standard echocardiographic techniques.

### 2.5. Laboratory Assessment

Routine laboratory investigations included: complete blood count (white blood cells, red blood cells, hemoglobin, thrombocytes), biochemical parameters (serum creatinine, glucose, and cholesterol panel). All laboratory tests were performed in certified hospital laboratories using standardized methods.

### 2.6. Assessment of Cardiovascular Risk Factors and Comorbidities

Traditional cardiovascular risk factors were evaluated in all patients, including arterial hypertension, diabetes mellitus (DM), body mass index (BMI), smoking status, and dyslipidemia. Comorbid cardiovascular disease included coronary artery disease (CAD).

### 2.7. Anticoagulant Therapy

The choice of anticoagulant therapy for the treatment of VTE was based on current clinical recommendations and individual patient characteristics. Long-term anticoagulation was performed using direct oral anticoagulants (DOACs) or low molecular weight heparin (LMWHs). Treatment selection considered bleeding risk, renal function, drug–drug interaction, and patient preference.

### 2.8. Statistical Analysis

Data analysis was performed using SPSS version 26.0 (IBM Corp., Armonk, NY, USA). Continuous variables are presented as mean ± standard error of the mean (M ± m), while categorical variables are expressed as absolute numbers and percentages. Group comparisons were conducted using Student’s *t*-test for continuous variables and the chi-square test for categorical variables. Univariate logistic regression analysis was used to identify variables associated with VTE occurrence. Variables with a significance level of *p* < 0.05 in univariate analysis were subsequently entered into a multivariate stepwise logistic regression model to identify independent predictors of VTE. Odds ratios (OR) and 95% confidence intervals (CI) were calculated. A two-side *p* value < 0.05 was considered statistically significant.

## 3. Results

### 3.1. Baseline Characteristic of the Study Population

A total of 116 patients with histologically confirmed DC were included in the analysis. The baseline demographic, anthropometric, and clinical characteristics of the study population are summarized in Table 1.

The mean age of the cohort was 53.1 ± 1.2 years, and all patients were female. Sixteen patients (13.8%) were older than 65 years. The mean BMI was 28.1 kg/m^2^. Dyslipidemia was presented in 26.7% of patients, arterial hypertension in 38.8%, coronary artery disease in 10.3%, and diabetes mellitus in 2.6%.

The majority of patients (93.9%) had stage II or III BC at diagnosis. Most patients (64%) received anthracycline-based CT regimens, including epirubicin or doxorubicin, in combination with cyclophosphamide, taxanes, trastuzumab, or other agents.

### 3.2. Incidence of Venous Thromboembolism

During the follow-up period, VTE occurred in 25 patients (21.6%). Based on VTE occurrence, patients were divided into two groups:

Group 1: patients who developed VTE (*n* = 25)

Group 2: patients without VTE (*n* = 91).

A comparison of baseline clinical, oncological, laboratory, and hemodynamic characteristics between the two groups is presented in Table 2.

Patients who developed VTE were significantly older than those without VTE (63.8 ± 1.9 vs. 50.2 ± 1.3 years, *p* < 0.01). Arterial hypertension was more prevalent in Group 1 compared with Group 2 (56.0 vs. 34.2%, *p* < 0.05), as was dyslipidemia (40.0 vs. 23.1, *p* < 0.05).

There were no significant differences between groups with respect to BMI, smoking status, CAD, or DM. Patients with VTE tended to have a poorer functional status, with ECOG performance ≥ 3 observed more frequently in Group 1, although this difference did not reach statistical significance.

Systolic and diastolic BP values, as well as heart rate, were comparable between groups. However, LV EF was significantly lower in patients with VTE compared with those without VTE (58.1 ± 1.2% vs. 61.8 ± 0.5%, *p* < 0.05), which may be explained by impaired LV contractility, particularly in patients with PE.

Patients who developed VTE have significantly higher levels of total cholesterol and fasting glucose compared with patients without VTE (*p* < 0.05 for both).

Additionally, Group 1 demonstrated significantly higher WBC counts, as well as lower RBC counts and hemoglobin level (*p* < 0.05 for all comparisons). Elevated WBC count and anemia are recognized contributors to thrombotic risk in oncology patients [11,12].

Anthracycline-containing CT regimens were used more frequently in patients without VTE (68.1%) than in those with VTE (48.0%); however, the cumulative anthracycline dose did not differ significantly between groups. HER2/neu-positive status and trastuzumab use were also more common in Group 2. No significant differences were observed regarding RT or endocrine therapy exposure (Table 3).

Surgical treatment was performed less frequently in Group 1; however, in two patients VTE developed within 30 days after surgery. According to available evidence, postoperative VTE risk in BC patients is considered relatively low, but perioperative events remain clinically relevant [13].

The type, timing, and treatment of VTE events are detailed in Table 4. DVT of the lower or upper extremities was the most frequent manifestation (48.0%), followed by PE alone (24.0%) and combined PE and DVT (20.0%). Catheter-related thrombosis and isolated upper extremity DVT were observed less frequently (8%).

**Figure 1:**
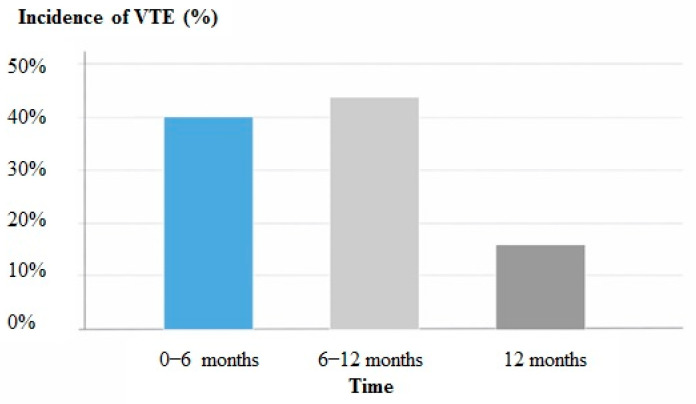
Temporal distribution of VTE in BC patients during follow-up.

VTE most commonly occurred within the first 12 months after BC diagnosis: 40.0% within the first 6 months and 44.0% between 6 and 12 months, while 16.0% occurred after one year (Figure 1). This temporal distribution reflects the period of highest thrombotic vulnerability during cancer treatment.

Varicose veins or a history of thrombophlebitis were presented in 40.0% of patients with VTE, suggesting an additional predisposing factor for thrombosis.

DOACs were used as long-term anticoagulant therapy in the majority of patients with VTE (76.0%), whereas LMWHs were prescribed in 24.0% of cases.

### 3.3. Predictors of Venous Thromboembolism

In univariate analysis, age ≥ 55 years, ECOG performance status ≥ 3, elevated glucose level, and decreased hemoglobin level were significantly associated with VTE development (*p* < 0.05 for all). No significant association was observed between the Khorana Risk Score and VTE occurrence.

Multivariate logistic regression analysis identifies three independent predictors of VTE: age ≥ 55 years (OR 1.74, 95% CI: 1.18 to 2.72), ECOG performance status ≥ 3 (OR 1.92, 95% CI: 1.29 to 2.12), and elevated glucose level (OR 2.21, 95% CI: 1.53 to 3.14).

### 3.4. Risk-Adapted Monitoring and Prevention Strategy

**Figure 2:**
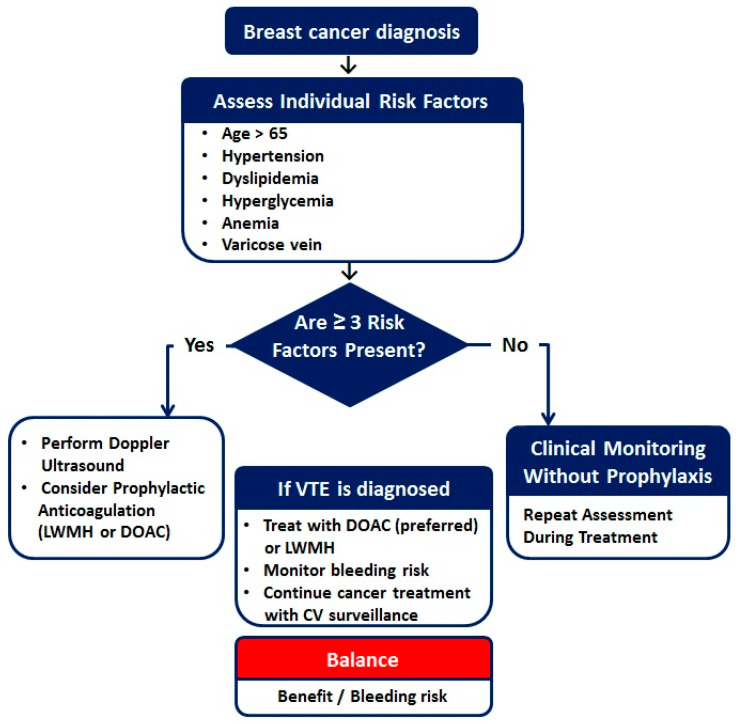
Risk-adapted algorithm for VTE monitoring and prevention in BC patients.

Based on the identified predictors, a risk-adapted algorithm for VTE monitoring and prevention in BC patients was proposed (Figure 2). This strategy integrates clinical, laboratory, and treatment-related factors to personalize thrombosis risk and prophylaxis, aiming to improve patient safety while minimizing bleeding risk.

#### Study Limitations

This study has several limitations, including its single-center, retrospective design, and relatively small sample size, which may limit generalizability. The absence of a standardized protocol of thromboprophylaxis and potential selection bias should also be acknowledged.

## 4. Discussion

CAT represents a major challenge in contemporary cardio-oncology, reflecting the complex interplay between malignancy, anticancer therapies, cardiovascular comorbidities, and systemic metabolic disturbances. Although BC has traditionally been classified as a tumor type with a relatively low thrombotic risk, the present study demonstrates that VTE is a frequent and clinically relevant complication in patients undergoing modern multimodal BC treatment [14].

These findings call into question conventional risk stratification paradigms and underscore the need for an integrated cardio-oncological approach to thrombosis prevention and monitoring.

In our cohort, VTE occurred in 21.6% of patients, with the highest incidence observed within the first 12 months after BC diagnosis. This temporal pattern is consistent with large population-based studies indicating that the peri-diagnostic and early treatment phase represent the period of maximal thrombotic vulnerability in cancer patients [15,16,17]. From a cardio-oncology perspective, this interval coincides with cumulative exposure to surgical intervention, systemic CT, RT, and targeted therapies, all of which may promote endothelial injury, inflammatory activation, and dysregulations of the coagulation cascade [18,19].

The high VTE incidence observed in this study likely reflects selection bias inherent to a cardio-oncology referral center, which manages a high-risk patient population with significant cardiovascular comorbidities. Consequently, these finding should be interpreted in the context of a clinically enriched cohort rather than a population-based BC sample.

Importantly, this early high-risk window overlaps with the phase of intensified cardiovascular surveillance in cardio-oncology practice, highlighting the necessity of integrating thrombotic risk assessment into routine cardiovascular monitoring during initial cancer treatment.

A key finding of the present study is that patient-related factors, rather than cancer-specific or treatment-related variables, were the primary determinants of VTE development. Advanced age emerged as a strong independent predictor, in line with previous reports and established clinical risk models, including the Geneva score [20]. Aging is associated with endothelial dysfunction, impaired fibrinolysis, and a higher burden of cardiovascular comorbidities, all of which amplify thrombotic risk in oncology populations.

Poor functional status (ECOG performance status ≥ 3) was independently associated with VTE occurrence. This likely reflects the combined effects of reduced mobility, frailty, systemic inflammation, and heightened cardiovascular vulnerability. Similar observations have been reported in large BC registries, where impaired performance status consistently predicted adverse thrombotic outcomes [21,22].

Of particular relevance to cardio-oncology is the identification of hyperglycemia as an independent VTE predictor. Metabolic dysregulation is increasingly recognized as a key contributor to thrombogenesis through mechanisms, involving endothelial dysfunction, oxidative stress, platelet hyperactivity, and activation of procoagulant pathways. In patients with BC, metabolic abnormalities frequently coexist with hypertension, dyslipidemia, and subclinical cardiovascular disease, forming a high-risk cardiometabolic phenotype that predisposes to CAT. These findings reinforce the concept that metabolic optimization should be considered an integral component of thrombosis prevention strategies in cardio-oncology practice.

Patients who developed VTE exhibited higher leukocyte counts and lower hemoglobin levels. Leukocytosis reflects systemic inflammation and neutrophil-driven prothrombotic mechanisms, including neutrophil extracellular trap, which has been increasingly implicated in the pathogenesis of CAT [11,13]. Anemia, particularly at moderate-to-severe levels, is a recognized thrombotic risk factor and may further exacerbate tissue hypoxia, endothelial dysfunction, and cardiovascular strain [12].

From a cardio-oncological perspective, these laboratory abnormalities should be interpreted not merely as oncological markers but as indicators of global cardiovascular and thrombotic risk, warranting closer surveillance and potentially earlier preventive interventions.

The Khorana Risk Score did not demonstrate predictive utility in this BC cohort, consistent with prior systematic reviews and meta-analyses [10]. Although widely applied in ambulatory oncology populations, the Khorana score appears insufficient for tumors traditionally classified as low risk, such as BC. This limitation is particularly relevant in cardio-oncology, where reliance on generalized risk models may result in under-recognition of patients with high-risk cardiovascular phenotypes.

Our findings support the growing consensus that tumor-agnostic risk scores should be complemented or replaced by individualized, cardio-oncology-oriented risk assessment frameworks incorporating functional status, metabolic parameters, inflammatory markers, and cardiovascular comorbidities.

In contrast, exposure to anthracyclines, trastuzumab, RT, or endocrine therapy was not independently associated with VTE in multivariate analysis. Although several anticancer therapies are known to affect endothelial function and coagulation pathways, current evidence does not support routine pharmacological thromboprophylaxis in unselected BC populations [8,22,23,24].

Nevertheless, treatment-related cardiovascular toxicity may indirectly influence thrombotic risk by impairing cardiac function or promoting vascular injury. The lower LV EF in patients with VTE, particularly those with PE, highlights the bidirectional relationship between cardiac dysfunction and thromboembolic events and reinforces the need for integrated cardiac and thrombotic monitoring.

DOACs were the predominant long-term anticoagulant therapy in this cohort, reflecting current guideline-supported practice. Accumulating evidence supports the efficacy and acceptable safety profile of DOACs in CAT, particularly in patients without gastrointestinal malignancies or high bleeding risk [25]. In BC patients, who generally exhibit a lower hemorrhagic risk profile, DOAC-based strategies appear especially feasible within a cardio-oncology framework.

Based on the identified predictors, we propose a risk-adapted algorithm for VTE monitoring and prevention that integrates age, functional status, cardiometabolic abnormalities, hematological parameters, and cardiovascular comorbidities. This approach aligns with the principles of modern cardio-oncology, emphasizing early risk stratification, personalized surveillance, and judicious use of preventive anticoagulation.

## 5. Conclusions

VTE represents a significant and often underestimated complication in patients with BC, particularly during early phases of treatment. Our findings emphasize that patient-related and cardiometabolic factors are central drivers of thrombotic risk and should be systematically addressed within cardio-oncology programs. Prospective, multicenter studies are warranted to validate these observations and to develop BC-specific, cardio-oncology-oriented risk models capable of guiding personalized thromboprophylaxis strategies.

## Figures and Tables

**Table 1:** Baseline demographic and clinical characteristics of patients with breast cancer.

Variable	Value of the Indicator (*n* = 116)
Baseline Characteristics	
Age, years	53.1 ± 1.2
Patients >65 years old, *n* (%)	16 (13.8)
Smoking, *n* (%)	2 (1.7)
BMI, kg/m^2^	28.1 ± 0.6
Dyslipidemia, *n* (%)	31 (26.7)
Coronary artery disease, *n* (%)	12 (10.3)
Diabetes mellitus, *n* (%)	3 (2.6)
Hypertension, *n* (%)	45 (38.8)

Abbreviations: BMI—body mass index. Data are presented as mean ± standard error of the mean or absolute number (%).

**Table 2:** Clinical, laboratory, and hemodynamic characteristics of patients with and without VTE.

Variable	VTE Group (*n* = 25)	Non-VTE Group (*n* = 91)	*p*-Value
Age, years	63.8 ± 1.9	50.2 ± 1.3	*p* < 0.01
BMI, kg/m^2^	29.3 ± 0.9	27.8 ± 0.8	NS
Hypertension, *n* (%)	14 (56.0)	31 (34.1)	*p* < 0.01
Coronary artery disease, *n* (%)	4 (16.0)	8 (8.8)	NS
Smoking, *n* (%)	1 (4.0)	1 (1.1)	NS
Diabetes mellitus, *n* (%)	2 (8.0)	1 (1.1)	NS
Dyslipidemia, *n* (%)	10 (40.0)	21 (23.1)	*p* < 0.05
Cancer stage, *n* (%)			
I	0	2 (2.2)	NS
II	19 (76.0)	66 (72.5)	NS
III	3 (12.0)	21 (23.1)	NS
IV	3 (12.0)	2 (2.2)	NS
ECOG performance status ≥ 3	6 (24.0)	14 (15.4)	NS
**Laboratory parameters**			
Creatinine, μmol/L	79.4 ± 3.3	71.8 ± 3.2	NS
Total cholesterol, mmol/L	6.1 ± 0.1	5.3 ± 0.3	*p* < 0.05
Glucose, mmol/L	6.4 ± 0.7	5.3 ± 0.2	*p* < 0.05
White blood cells, ×10^9^/L	6.8 ± 0.7	5.1 ± 0.3	*p* < 0.05
Hemoglobin, g/L	114.7 ± 3.1	126.2 ± 3.2	*p* < 0.05
Red blood cells, ×10^12^/L	4.1 ± 0.1	4.5 ± 0.1	*p* < 0.05
**Hemodynamics**			
Systolic BP, mm Hg	123.6 ± 3.2	128.1 ± 2.0	NS
Diastolic BP, mm Hg	80.6 ± 12.0	87.4 ± 1.6	NS
Heart rate, beats/min	85.2 ± 2.8	87.2 ± 2.0	NS
Left ventricular ejection fraction, (%)	58.1 ± 1.2	61.8 ± 0.5	*p* < 0.05

Abbreviations: VTE—venous thromboembolism; BMI—body mass index; ECOG—Eastern Cooperative Oncology Group; BP—blood pressure; NS—not significant.

**Table 3:** Cancer treatment of BC patients.

Variable	VTE Group (*n* = 25)	Non-VTE Group (*n* = 91)	*p*-Value
Anthracycline-based chemotherapy, *n* (%)	12 (48.0)	62 (68.1)	*p* < 0.05
Cumulative anthracycline dose mg/m^2^	224.5 ± 11.2	227.5 ± 16.8	NS
Trastuzumab, *n* (%)	5 (20.0)	31 (34.1)	*p* < 0.05
Radiation therapy, *n* (%)	8 (32.0)	30 (33.0)	NS
Endocrine therapy, *n* (%)	4 (16.0)	18 (19.8)	NS
Surgical treatment, *n* (%)	10 (40.0)	58 (63.7)	*p* < 0.05

**Table 4:** Characteristics of VTE and risk factors in patients with BC.

Variable	Value, *n* (%)
**Type of VTE**	
Pulmonary embolism (PE)	6 (24.0)
Lower extremities deep vein thrombosis (DVT)	12 (48.0)
PE + DVT	5 (20.0)
Upper extremities DVT (UE DVT)	1 (4.0)
Catheter-related DVT (port-associated)	1 (4.0)
**Time of VTE occurrence after BC diagnosis**	
0–6 months	10 (40.0)
6–12 months	11 (44.0)
>12 months	4 (16.0)
D-dimer, ng/mL	2261.1 ± 492
**Risk factors for VTE**	
Surgical treatment within 30 days	2 (8.0)
Varicose veins	10 (40.0%)
**Anticoagulant therapy**	
Low-molecular-weight heparin	6 (24.0)
Direct oral anticoagulants	19 (76.0)

Abbreviations: BC—breast cancer; VTE—venous thromboembolism; PE—pulmonary embolism; DVT—deep vein thrombosis; UE DVT—upper extremities DVT.

## Data Availability

Data are available upon request.

## Abbreviations

The following abbreviations are used in this manuscript:

BC	Breast cancer
VTE	Venous thromboembolism
DVT	Deep vein thrombosis
PE	Pulmonary embolism
PH	Pulmonary hypertension
CAT	Cancer associated thrombosis
CCT	Comprehensive antitumor therapy
CT	Chemotherapy
RT	Radiation therapy
BMI	Body mass index
CAD	Coronary artery disease
DM	Diabetes mellitus
DOAC	Direct oral anticoagulants
LMWH	Low molecular weight heparin
